# Older Adults’ Biobehavioral Fall Risks Were Affected by the COVID-19 Pandemic: Lessons Learned for Future Fall Prevention Research to Incorporate Multilevel Perspectives

**DOI:** 10.1093/geroni/igac033

**Published:** 2022-06-11

**Authors:** Hiroko Kiyoshi-Teo, Shigeko (Seiko) Izumi, Sydnee Stoyles, Siobhan K McMahon

**Affiliations:** School of Nursing, Oregon Health and Science University, Portland, Oregon, USA; School of Nursing, Oregon Health and Science University, Portland, Oregon, USA; School of Nursing, Oregon Health and Science University, Portland, Oregon, USA; School of Nursing, University of Minnesota, Minneapolis, Minnesota, USA

**Keywords:** Biobehavioral, Health behaviors, Injury prevention, Pandemic, Socioecological framework

## Abstract

**Background and Objectives:**

Examining the impact of coronavirus disease 2019 (COVID-19) pandemic on fall risks may provide insight into how multilevel factors as described in National Institute of Nursing Research's (NINR’s) draft strategic plan can guide future fall prevention research. This article describes the affect of COVID-19 on fall risks from the perspective of older adults who live in assisted living facilities (ALFs), and explores the needs and approaches to implement fall prevention interventions at individual, social, community, and policy levels.

**Research Design and Methods:**

Exploratory survey study. Participants from a fall prevention study at 2 ALFs in Oregon were invited to the study. Survey questions asked about COVID experience, and changes in fall risks and day-to-day activities in Spring 2020. Quantitative responses were analyzed using descriptive statistics and Cohen’s *d* effect sizes. Qualitative responses were analyzed using conventional content analysis.

**Results:**

Thirteen participants (age: *M =* 87.08, standard deviation = 6.52) responded. More participants reported feeling unsteady compared to pre-COVID data (38% vs. 62%), while the proportion of those worried about falling remained the same at 38%. Participants reported negligible decreases in importance of fall prevention and small decreases in confidence of fall prevention (Cohen’s *d* = −0.13 and −0.21, respectively). The themes related to the affect of COVID on fall risks were: *not to worry about fall risks but be cautious* and *physical activity is important, but it’s hard during COVID*. Impact of COVID on day-to-day activities were: *varying degrees of concern for COVID, lack of social and community support*, and *finding unique ways to cope with COVID.*

**Discussion and Implications:**

These individual-level perspectives suggest that older adults were at increased risk for falling. Results exemplify the influence of broader-level factors (e.g., social, community, and policy) on individual biobehavioral factors (e.g., fall risks and health behaviors), and illustrate the value of examining multilevel factors in future fall prevention research.

**Translational Significance:** Coronavirus disease 2019 lockdown measures were necessary for protecting individuals from the fatal infection. However, these restrictions had affect on other aspects of people’s health. Our study explored experiences of older adults in assisted living facilities during the pandemic and showed how the lockdown affected their daily life and fall risks on the multiple levels. Findings urge the researchers to expand fall prevention interventions from solely focusing on individual level to multilevel strategies including socioecological perspectives. A few examples include facilitating social connections to encourage fall prevention actions, and integrating and normalizing fall prevention strategies into existing community programs and activities.

One of the major adverse impacts of coronavirus disease 2019 (COVID-19) prevention policies, such as physical distancing, is increased fall risk in older adults (Hoffman et al., 2022). Physical distancing, often called social distancing, is an infection control measure to limit the spread of the COVID-19 virus. While important for infection control, physical distancing measures resulted in physiological changes such as decreased activity levels and physical functioning, and increased fall risks during the pandemic (Hoffman et al., 2022; Sands et al., 2020; Sepúlveda-Loyola et al., 2020; Stockwell et al., 2021). However, there is a lack of research that examines the affect of COVID prevention measures on other fall-risk factors, such as behavioral and environmental aspects of fall prevention (World Health Organization, 2007).

Older adults living in Oregon assisted living facilities (ALFs) experienced COVID lockdown early in the pandemic in February 2020 (State of Oregon, 2020). ALFs offer structured, supportive care for housekeeping, meals, and medications (National Institute of Aging, 2017). The lockdown measures mandated residents to confine to their rooms, pause their social activities, including dining and other gatherings, and maintain strict visitation restrictions (i.e., limited to essential health care staff and friends or family members at end-of-life stages only).

These restrictions, while essential, have aggravated the serious public health problem of falls among older adults especially among ALF residents (Hoffman et al., 2022; Nania, 2021). Approximately 30% of residents experience a fall in 90-day-period (Carder et al., 2017); however, fall prevention research in ALFs is largely limited (Cameron et al., 2010). Residents of ALFs have complex health management needs: more than 75% have at least two chronic illnesses; almost 40% require assistance with three or more activities of daily living (Caffrey et al., 2012). In addition, physical activity levels for ALF residents are very low; reported average time spent walking for any purpose is 0.5 hour per day (Bootsman et al., 2018). Evidence-based interventions for fall prevention exist (Gillespie et al., 2012); however, recommendations alone have not made significant affects on fall rates across older adult populations, including those living in ALFs (Harris-Kojetin & Sengupta, 2018).

Many residents have moved to ALFs for their supportive environment (physical and social) including systems that help people maintain active and healthy lifestyles, for example, minimization of fall risks. There are handrails in hallways and bathrooms for stability. Chairs are situated around communal areas so that residents can rest as needed. ALFs often offer in-person exercise classes multiple times a week. Meals are encouraged to be taken in the communal dining room. Trips to dining rooms are a major event during the day with the excitement of food and opportunity to interact and develop social relationships with neighbors. Social activities such as bingo games and movie nights, and excursions to shopping centers also help to build residents’ social community within ALF.

A socioecological lens is essential to create effective interventions to improve health outcomes (National Institute of Nursing Research, n.d.). While interventions to address biological fall risks are well studied, there’s paucity of research on fall prevention strategies that target health behavior change, environmental (physical and social), or community factors. Therefore, the purpose of this study is to examine how the COVID-19 lockdown may have affected biological, behavioral, and environmental fall risks in older adults living in ALFs. In this article, we first provide the background context of this study by briefly explaining the parent fall prevention intervention study. We then present our study methods and findings to explore older ALF residents’ experience of COVID on fall risks. Finally, we explore how future fall prevention intervention studies can address fall risks at individual, social, community, and policy levels based on the learning from this study.

## Background

We began the parent fall prevention study in (January 2019–April 2020) as a pilot study to evaluate the affect of fall prevention care management intervention integrated with motivational interviewing on reducing fall risks (*n* = 25; citation of X-blinded). Residents from two ALFs in Oregon, United States in urban settings and had residential capacities of 105 and 75 participated in the study. Participants’ inclusion criteria for the parent study were: high fall risk (i.e., had a fall in the past year or had multiple risk factors for a fall) and had a Montreal Cognitive Assessment (MOCA) Score of ≥15 (Nasreddine et al., 2005). Intervention consisted of 6 weekly 1-hour in-person visits for care management and motivational interviewing. We found improvement in fall risks such as fear of falling (Kempen et al., 2008), fall prevention behaviors (Clemson et al., 2003), and the level of confidence to prevent falls (Kiyoshi-Teo et al., 2019). The study was not designed to capture the affect of intervention on fall rates, but three individuals reported experiencing at least one fall within a year prior to joining the parent study, and one individual had a fall during the parent study.

## Method

### Design and Study Procedures

This is a cross-sectional survey study conducted in May 2020 during the COVID-19 pandemic. This study was conducted as a follow-up to the parent fall prevention study. Twenty-five study participants who completed the parent study were invited to participate via telephone and postal mail. All participants were eligible to participate in this survey study if they were still residing in ALFs and emotionally, physically, and cognitively well-enough to participate in the study based on research team’s judgement at the time of recruitment. Oregon Health & Science University Institutional Review Board approved the study.

The survey questions were administered via telephone. Telephone surveys were audio-recorded and transcribed. Paper surveys were mailed to those we were unable to reach by telephone with prestamped return envelope. The telephone survey took approximately 30 min to complete.

### Survey Questions

The survey included eight closed-ended and three open-ended questions. To capture the overall health of the participants, we asked: “How would you rate your general health?” (*excellent, very good, good, fair, and poor*). We included questions to address participants’ COVID exposure and experiences such as “have you been diagnosed with COVID?” (*yes, no*); “Do you know anyone who has COVID?” (*yes, no*); and “How concerned are you about COVID?” (*not at all concerned, somewhat concerned, very concerned, and not sure*).

We used validated quantitative measures to capture participants’ perceptions of fall risks such as unsteadiness, worry about falling, and importance and confidence to prevent themselves from falling. Centers for Disease Control and Prevention (CDC) fall risk assessment questions (CDC, 2019) “do you feel unsteady when standing or walking?” (*yes*, *no*) were used to assess physiological balance experienced by participants, and “do you worry about falling?” (*yes*, *no*) was used to assess behavioral fall risks. Self-reported unsteadiness and worry about falling are indicative of increased risk for falling (Eckstrom et al., 2021).

The level of importance and confidence scales are commonly used to gauge individuals’ potential for positive behavior change (Miller & Rollnick, 2012). Thus, participants were asked to rate the importance of fall prevention and their confidence to prevent falls. Both questions were rated from 0 to 100. A rating of 100 indicated high importance or confidence.

Open-ended questions were used to capture themes not measured by closed-ended questions, and to better understand participants’ responses to the closed-ended questions. The question that captured participants’ perceptions of the affect of COVID on fall risks was: “How do you think your chances of falling have changed for you with this COVID-19/Coronavirus pandemic?” If a participant’s response was brief, we asked them to elaborate. To broadly capture participants’ overall changes in day-to-day activities due to COVID, we asked “How have things changed for you with COVID-19/Coronavirus pandemic?” We asked this question because fall risks are closely connected with daily activities (Clemson et al., 2003).

Finally, we asked “what things have been helpful for you right now to get through this time?” to address participants’ resiliency with COVID. Resiliency is one of the key concepts to understand individual’s strengths and realizing potential in wellness (McMahon & Fleury, 2012). During COVID, resiliency may include strategies to cope with lack of social connections or to minimize the negative impact of social isolation, which is known to increase falls (Pohl et al., 2018).

### Data Analysis

Responses to the closed-ended questions were analyzed in R (R Core Team, 2020) to provide descriptive statistics. Cohen’s *d* effect sizes for importance and confidence in preventing falls were calculated using the *effsize* package (Torchiano, 2020). Responses from open-ended questions were analyzed by conventional content analysis approach (Hsieh & Shannon, 2005; Miles et al., 2018). Conventional content analysis is suited to describe a phenomenon by allowing categories and names for categories to emerge. Exact words from the text capturing key thoughts or concepts are noted. Three authors, all registered nurses with research doctorates and expertise in care of older adults (H. Kiyoshi-Teo, S. K. McMahon, and S. Izumi), participated in the analysis until the consensus was achieved. The final interpretation of the data was conducted by examining both the quantitative and qualitative data.

## Results

### Participants’ Characteristics

Of the 25 contacted individuals, 13 participants participated in the study (11 females and two males). Ten individuals completed telephone interviews, and three participants returned a paper survey. The mean age of the participants was 87.08 (standard deviation [*SD*] = 6.52), and the mean MOCA score was 21.77 (*SD* = 3.68). Three quarters of participants indicated their general health was “good” to “very good.” None of the participants reported being diagnosed with COVID. Only 31% of respondents reported being very concerned about COVID. One participant reported a fall without injury within 3 months of participating in the study.

Sixty-two percent of participants reported feeling unsteady when standing or walking, an increase compared to pre-COVID study exit data from the parent study (38%, Table 1). The proportion of participants worried about falling remained the same (38%) comparing pre-COVID study exit data and data collected from this study. Participants reported a negligible decrease of their belief in the *importance* of fall prevention (Cohen’s *d* = −0.13, 95% confidence interval [CI] [−1.26, 1.00]) and a small decrease in their *confidence* to prevent falling during COVID lockdown compared to before COVID (Cohen’s *d* = −0.21, 95% CI [−0.89, 0.47]).

**Table 1.** Perceptions of Fall Risks Pre-COVID and COVID Period

Fall-risk perceptions	Pre-COVID (parent study)^a^		COVID (survey study)	
	*N* (%)	Mean ± *SD*	*N* (%)	Mean ± *SD*
Feels unsteady while standing or walking				
Yes	5 (38%)		8 (62%)	
No	7 (54%)		4 (33%)	
Unsure	1 (8%)		1 (8%)	
Worries about falling				
Yes	5 (38%)		5 (38%)	
No	6 (46%)		5(38%)	
Unsure	2 (15%)		3 (24%)	
Level of importance to prevent falling^b^		86.83 ± 26.98		83.18 ± 17.36
Level of confidence to prevent falling^b^		72.55 ± 21.03		70.73 ± 17.92

*Notes: N* = 10–13 (sample sizes differ slightly for each of the analyses due to missing data). COVID = coronavirus disease; *SD* = standard deviation.

^a^Data were collected postintervention in the parent study.

^b^Rating from scale of 0 to 100. One hundred represents most important/confident.

### Impact of COVID-19 on Fall Risks

Two themes arose from the responses to the question “How do you think your chances of falling have changed for you with the COVID-19/Coronavirus pandemic?” They were (a) not to *worry* about fall risks but be *cautious* and (b) physical activity is important, but it’s hard during COVID.

#### Not to worry about fall risks but be cautious

Several participants emphasized that their fall risks are not different because of the pandemic and said “people fall sometimes” normalizing fall risks, and explained that it’s common sense to prevent falls. Some even described how the pandemic was protective for their fall risks. They explained that because they were restricted to their own room their fall risk would be less during the pandemic. However, they remarked that they are cautious to keep them from falling despite perceived lower risk for falls. For example, one participant Ron (study assigned name for anonymity; age 97) recently fell in his room, and the staff had to come to get him up.

I don’t worry about it (falling risks), but I’m pretty concerned about it ‘cause I fell too many times … I wouldn’t say it (COVID) changed my chances of falling. But being cooped up in here, I don’t think I have any more chance of falling, maybe even less because I watch myself more closely now than I used to. … I can’t imagine myself sitting here for days doing nothing but twiddling my thumbs, that ain’t me. I got to be able to do stuff. So I’m more careful now than I was before this thing (COVID lockdown) happened … .

Similarly, another participant (Kate; age 77) acknowledged her lack of balance, but feels that it is the same as before the pandemic. She described how she doesn’t “stress about” her fall risks and worry, but explains how she is being thoughtful about staying safe by being physically active and using the walker. She stated:

I just keep moving around my room and trying to do some stretching and things, but not as much as I like to … I feel unsteady, about the same (as compared to before the pandemic) … I don’t trust myself walking without my walker, I am using more walker right now … I guess it (fall risks) is in the back of my mind, but it’s not something I stress about. I’m very, pretty much active, it’s just a matter of walking around.

#### Physical activity is important, but it’s hard during COVID

Some reported their activity level went down because they had to stay in their room, while others described how they were able to maintain their physical activities. One participant (Clara; age 85) described how physical activity decreased for her, and how that led to feeling weaker. She said:

I spend a lot of time laying on my bed, because that’s the only place that is more comfortable. I tried to go down to the mailbox, it gives me a chance to walk. But I go down every couple of days or something … I use the walker less because there’s nowhere to go. I only go to the bathroom or the bedroom … Yeah, I definitely do (feel I am getting weaker) because I’m not walking near what I used to.

Conversely, participant Ron describes that he was exercising more because exercise was his way to cope with COVID lockdown. All participants mentioned their intentions to maintain their physical activity at varying levels, but these activities were mostly about walking and limited to their rooms. They did not mention any resources such as YouTube videos or TV program, to guide their exercise.

### Impact of COVID-19 on Day-to-Day Activities

The question “How have things changed for you with COVID-19/Coronavirus pandemic?” yielded broader themes about their everyday experiences during the pandemic. We captured their perceptions about COVID and COVID-related policies, and social and community changes that directly or indirectly affected their perceptions of fall risks. Three themes were identified: (a) varying degrees of concerns vary for COVID; (b) lack of social and community support; and (c) finding unique ways to cope with COVID.

#### Varying degrees of concerns vary for COVID

Some described a sense of security from infection because they were following strict quarantine policies, and indicated a positive outlook. For example, one participant (Kim; age 87) said “I am concerned about it (COVID), but I’m not overly concerned. I’ve managed to make it this far. I just go on but I wear a mask.” Conversely, another participant (Jane; age 83) was concerned despite following all the rules and said:

I’m really concerned. I’ve been following all the rules. Everything is done in my room. I think it’s been eight weeks, and I haven’t been out of the room that I can remember. I’ve only been out of my room once and that was to go to the doctor.

Although Jane was following the rules, she describes that these were rules that she obtained from watching the news on TV. She didn’t feel like the facility gave much guidance, thus feeling frustrated that she was not well-supported by her ALF community.

#### Lack of social and community support

Many participants were experiencing loneliness due to lack of social connection with important others such as family, friends, and neighbors. For example, one participant (Mary; age 91) described the loneliness she feels due to lack of social support from her family, and the frustration of not being able to proactively address this social isolation by going out herself.

I miss being able to get out of the room and talking to people seeing other people. Yeah, if it’s (COVID-19 prevention precautions) necessary, it’s necessary. Yeah … one thing is that my children can’t visit me. Yeah. And I can’t go out and I do feel stuck in the room.

For others, lack of social support meant potential gaps in their health maintenance activities such as managing medications and prescribed diet. A participant (Ron) said that his daughter was no longer able to come to his room to set up his medications, and he needed to problem-solve how he was going to take his medications. Another participant (Jane; age 83) who is diabetic described how changes in food and loneliness affected her appetite during the pandemic. To make matters worse, she was no longer able to receive glycemic-friendly food that her family prepares for her. Jane said:

Their food has gotten worse … a lot of times I throw it away and I don’t eat it … I do try and eat the night meal because it’s so long until breakfast the next morning … Since all the virus stuff, (food is) not so good. And that’s bad when you’re in your room and there’s no social contact (laughs).

Because she was not able to eat the food that was delivered to her room, she ended up snacking, contributing to more frequent episodes of hyperglycemia during the pandemic.

#### Finding unique ways to cope with COVID

From the responses to the question “what things have been helpful for you right now to get through this time?” we identified how participants were coping with COVID-related restrictions in various ways. Some were reflective of their past life, while others found ways to pass time, find meaningful activities, and find ways to connect with others during the lockdown. For example, one participant (Jane) described how she compared herself to other difficult times in her life, and had identified activities to pass time “I’ve done a lot of tougher things. I’ve got my computer and my TV. I always have a book to read.” Another participant, Kim, described how she found meaning in helping others through acts of kindness. She had decided to collect flowers from the ALF property and had been delivering them to other residents. Another participant, Ron, coped by focusing on things he had control over, and was exercising more. Other participants said talking to others on the phone, video-chat, and text messaging had been helpful to cope with the pandemic.

## Discussion

This study was conducted to explore older adults’ perceptions of fall risks approximately 3 months into the COVID-19 lockdown at ALFs. We found that participants experienced increased unsteadiness, decreased physical activity, and confidence to prevent falls, but these did not translate to increased worry or importance to prevent falls. In describing the impact of COVID, participants stated how lockdown made it challenging to maintain their physical activity levels, social connections with family and friends, and access to societal services and resources to maintain their health. Prior studies indicate that self-reported unsteadiness and decreased physical activity are related to increased fall risks (Donoghue et al., 2017; Hoffman et al., 2022). Decreased confidence is negatively associated with positive health outcomes of interest (Bertholet et al., 2012). Thus, our findings suggest a potential increase in biobehavioral fall risks in older adults in ALFs during the pandemic. Our findings are similar to Hoffman et al.’s national online survey (*n* = 2,006) that found increases in unsteadiness and fear of falling, and decreases in physical activity, physical condition, and social isolation (2022).

Our study was unique in that we assessed the affect of COVID on day-to-day activities to broadly capture themes potentially related to fall risks. One of the important findings was that there was a lack of socioecological supports for ALF residents, in particular, to maintain physical activity and social connections during the pandemic. There could have been community support to encourage physical activities through TV exercise programming, or support to facilitate social connections by offering instrumental support with technology so that residents can connect with important others. However, ideas and strategies to foster social connections were limited for older adults (CDC, 2021). Strategies to foster children’s social and learning development during COVID restrictions (CDC, 2022) may offer suggestions for older adults as well. For example, creation of covered and heated outdoor spaces with better airflow to connect with others. Limiting the number of close contacts by creating cohorts of residents to maintain the minimal social connection while minimizing COVID spread risk. Additionally, staggered times for people to use communal resources by using a reservation-based system for exercise rooms or equipment could have been instituted. These strategies would not only reduce fall risks but minimize the negative impacts of social isolation (Sepúlveda-Loyola et al., 2020). Environmental support systems are essential for individuals and society to maintain wellness (Stokols, 1992). The urgency and necessity to focus on COVID infection control may have limited the capacity of ALFs to address other health promotion needs such as fall prevention, especially in the early stages of the pandemic.

## Limitations

The study was an exploratory cross-sectional survey administered at the beginning of the pandemic. We had a small sample that limited the view of the pandemic experience for ALF residents on fall risks. We did not examine the affect of COVID-19 on fall rates. Recruitment and retention of older adults in fall prevention studies are challenging (Nyman & Victor, 2011). To maximize enrollment, we used both telephone and mail for recruitment and data collection. We had limited representation of male or non-White perspectives. Survey studies are prone to social desirability bias (Fisher & Katz, 2000); however, open-ended and closed-ended questions provided diverse perspectives to understand the impact of the pandemic in relation to fall prevention.

## Future Directions

**Figure 1.**
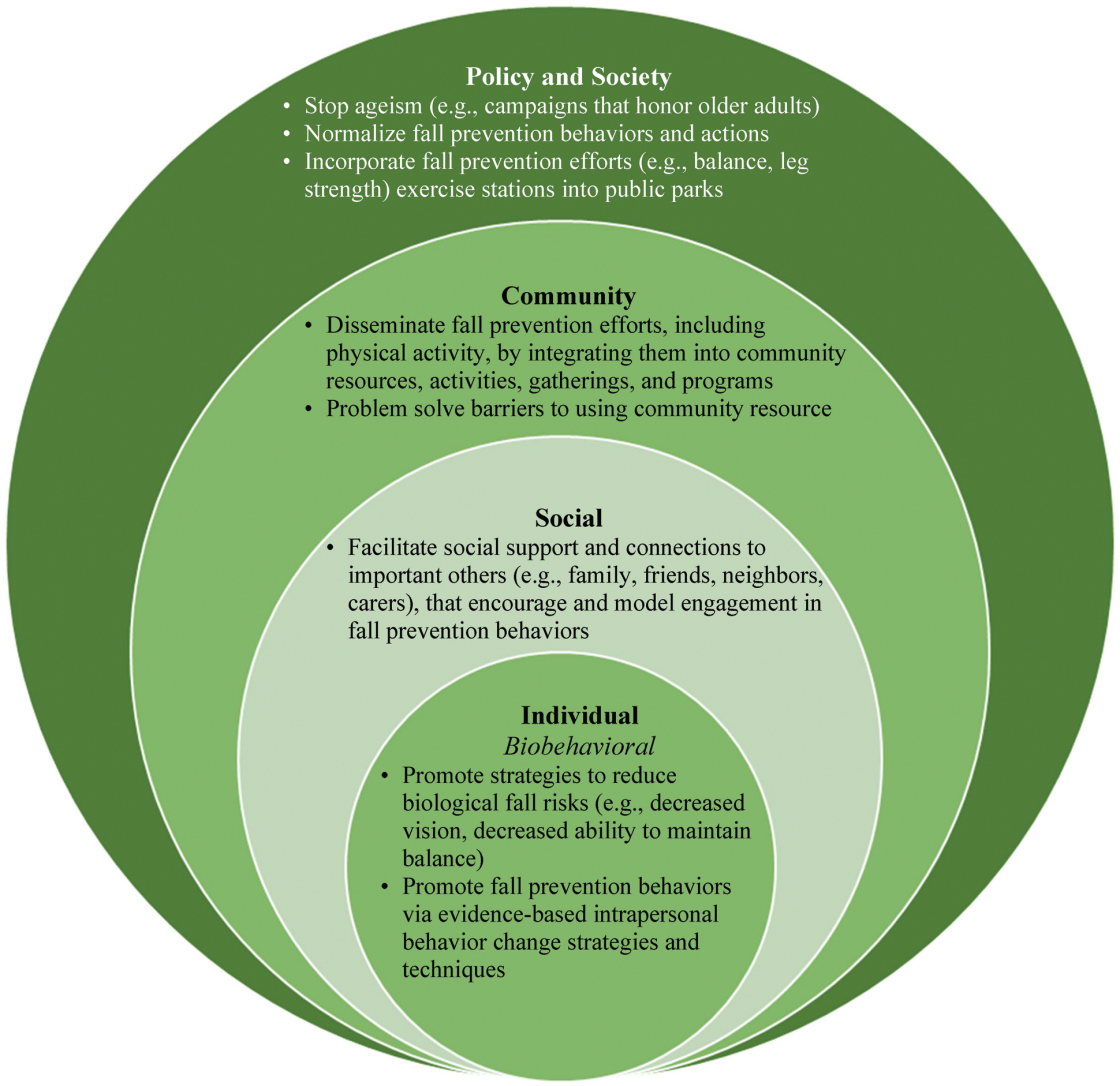
Multilevel strategies for fall prevention.

Despite the limitations, this study provided some insights: (a) multilevel factors affected individual’s biobehavioral fall risks; (b) socioecological supports were insufficient to address pandemic-related changes to support older adults to continue their engagement in fall prevention, especially with maintaining physical activity and minimizing the negative impact of social isolation. Thus, we applied multilevel perspectives (National Institute of Nursing Research, n.d.) to identify how we can integrate biobehavioral perspectives with different levels of the socioecological factors for future fall prevention research (Figure 1). We have identified a starting list of needs and potential approaches:

- Approaches to fall risk factor(s) as chronic conditions (National Council on Aging, 2021a) and examine strategies such as care management (Kiyoshi-Teo et al., 2021; Reuben et al., 2017) and use patient-centered study outcomes such as quality of life (Bjerk et al., 2017).
- Individual-level strategies may include further testing and development of behavior change strategies such as motivational interviewing (Arkkukangas & Hultgren, 2019; Kiyoshi-Teo, Northup-Snyder, et al., 2019a,b), goal setting (Reuben et al., 2017), intrapersonal and interpersonal behavior change techniques (McMahon et al., 2019) to help older adults maintain fall prevention behavior.
- Social level strategies to encourage engagement in fall-reducing behaviors through facilitating social connections (National Institute on Aging, 2021; Sepúlveda-Loyola et al., 2020), because one important negative impact of social isolation is a decrease in physical activities.
- Community-level strategies might include identifying barriers and facilitators affecting older adults’ uptake of community resources and creating an environment in which fall prevention and physical activity community resources are well disseminated, implemented, and promoted (McMahon et al., 2019). Repeated exposures to empowering messaging about fall prevention to older adults and people interacting with older adults (i.e., how proactive management of fall risks are helpful, health can be improved despite age) can be a powerful strategy to improve the readiness to engage in fall prevention when fall prevention resources become available to them.
- Societal and policy-level strategies include efforts against ageism. For example, campaigns that honor older adults, such as those led by National Council of Aging (National Council on Aging, 2021b) and intergenerational social interactions (Burnes et al., 2019).

## Conclusion

Our exploratory study examined how the COVID-19 pandemic affected fall risks in older adults who were experiencing lockdown in ALFs. We found that lockdown affected participants’ perception of fall risks. Participants experienced increased unsteadiness, and decreased physical activity. They were less confident in their ability to prevent a fall despite being confined to their rooms in lockdown. Participants voiced that lockdown also significantly limited personal social activities and access to societal resources. There were opportunities for community-level support to maintain physical activity levels and for social connections. Results exemplify the influence of broader-level factors (e.g., lockdown policy, lack of social and community support for fall prevention) on individual biobehavioral fall risk factors (e.g., fall risks and health behaviors). Lessons learned from this study inform how multilevel strategies are essential to support individuals to enable and continue their engagement with fall prevention.
